# Differential impact of circulating tumor cells on disease recurrence and survivals in patients with head and neck squamous cell carcinomas: An updated meta-analysis

**DOI:** 10.1371/journal.pone.0203758

**Published:** 2018-09-07

**Authors:** Jae-Keun Cho, Gil Joon Lee, Hae-Dong Kim, Uk Yeol Moon, Min-Ji Kim, Seonwoo Kim, Kwan-Hyuck Baek, Han-Sin Jeong

**Affiliations:** 1 Department of Otorhinolaryngology-Head and Neck Surgery, Inje University Ilsan Paik Hospital, Inje University School of Medicine, Goyang, Republic of Korea; 2 Department of Otorhinolaryngology-Head and Neck Surgery, Kyungpook National University Chilgok Hospital, Kyungpook National University School of Medicine, Daegu, Republic of Korea; 3 Department of Otorhinolaryngology-Head and Neck Surgery, Samsung Medical Center, Sungkyunkwan University School of Medicine, Seoul, Republic of Korea; 4 Statistics and Data Center, Research Institute for Future Medicine, Samsung Medical Center, Seoul, Republic of Korea; 5 Department of Molecular and Cellular Biology, Samsung Biomedical Research Institute, Sungkyunkwan University School of Medicine, Suwon, Republic of Korea; University of Calgary Cumming School of Medicine, CANADA

## Abstract

**Purpose:**

The prognostic impact of circulating tumor cells (CTC) on disease recurrence, progression and survivals in patients with head and neck squamous cell carcinoma (HNSCC) has not been adequately described. The objective of this study was to determine the impacts of the presence of CTC on loco-regional recurrence and survival of HNSCC patients by conducting a systematic review and meta-analysis.

**Methods:**

A comprehensive search for articles published between 1990 and 2016 was conducted and data from these studies were extracted, using the MEDLINE, Cochrane Library, and EMBASE databases. The main outcomes were overall survival (OS) and recurrence-free survival (RFS) of HNSCC patients. Pooled hazard ratio (HR) and 95% confidence intervals (95%CI) were calculated using the random effect model for outcomes. The quality of the studies, heterogeneity and publication bias were assessed with the appropriate statistical methods.

**Results:**

Six eligible studies with 429 patients were identified. The presence of CTC was significantly associated shorter RFS (HR = 4.88 [95%CI: 1.93–12.35], *P* < 0.001). However, it could not predict patients’ OS (HR = 1.92 [95%CI: 0.93–3.96], *P* = 0.078). The following analyses using univariable values of each study also made the similar results (HR = 1.70 [95%CI: 0.83–3.45] for OS, HR = 3.79 [95%CI: 2.02–7.13] for RFS). Heterogeneity and publication bias were not significant, except one enrolled study.

**Conclusions:**

The presence of CTC is not a significant prognostic indicator for OS of patients with HNSCC, although it could reflect the outcomes of loco-regional disease.

## Introduction

Loco-regional control for head and neck squamous cell carcinomas (HNSCC) has been improved with advances in new diagnostic modalities [1] and radiation techniques [2], refinements of surgical procedures [3] and the introduction of multi-modal treatments.[4–6]

However, the frequency of distant metastasis and the overall survival rate have not been significantly improved in the last 20 years, particularly for the advanced stage HNSCC.[7,8] Tumor metastasis to vital organs is a factor critical to cancer mortality in patients with HNSCC.[7] Therefore, early identification of a subset of patients prone to future distant metastasis is important to improve the outcomes of patients with HNSCC.

Dissemination of tumor cells into blood circulation is an important step in the initiation of tumor metastasis.[9] Circulating tumor cells (CTCs) play an important role in early diagnosis, prognosis prediction, and selection of treatment modalities in many types of cancer.[10–12] Clinically, CTC measurement has been applied in breast cancer [11], lung cancer [13], hepatocellular carcinoma [14], prostate cancer [12], and colorectal cancer.[10] The prognostic value of CTCs in HNSCC has also been studied;[15,16] however, whether the presence of CTC is associated with different endpoints of survival remains controversial.

Meta-analysis is a statistical technique that combines the findings from independent studies, with a benefit of having a higher statistical power and more robust point estimate.[17,18] Recently, results of two meta-analyses about the prognostic value of CTCs in HNSCC have been published.[15,16] One study has indicated that CTC-positive rate in groups with disease progression (recurrence/metastasis) is significantly higher than that in patients without disease progression.[15] On the other hand, the other study has reported that the presence of CTC is significantly associated with shorter disease free survival, but not with disease progression free or overall survival.[16]

Given these conflicting results, we aimed to determine the impacts of the presence of CTC on recurrence (relapse after the curative treatment) free (= disease free) survival (RFS), progression (metastatic tumor progression) free survival (PFS) and overall survival (OS) by conducting a systematic review and meta-analysis.[19–21] Results of our analysis will elaborate the clinical significance of CTC in the settings of loco-regional disease or distant metastasis in patients with HNSCC.

## Materials and methods

### Search strategy

This study was performed according to the recommendations of the Preferred Reporting Items for Systematic Reviews and Meta-Analyses (PRISMA) 2009 guidelines (S1 Table).[22] We systematically searched clinical studies published prior to Dec., 2016 from Jan., 1990 in MEDLINE, Cochrane Library, and EMBASE databases. Potentially relevant studies were identified using the following key words: [circulating tumor cell(s)], in combination with [head and neck cancer], [carcinoma of the head and neck], or [squamous cell carcinoma of the head and neck] (S2 Table). In addition, reference lists of retrieved articles were screened manually to identify additional eligible studies. No language restriction was imposed. Due to the paucity of relevant literatures on publication database, we also searched the meeting abstracts and short reports / letters. The eligibility of these studies was decided through comprehensive reviews and discussions with multiple researchers (JKC, GJL, MJK, SK and HSJ).

### Study selection

The inclusion criteria for these studies were as follows: (i) the enrolled patients had a histological diagnosis of HNSCC, (ii) CTCs were detected in peripheral blood samples by various methods, and (iii) information about the association between CTC status and clinical outcomes (disease recurrence, metastatic tumor progression and death from any cause) was available. Studies were excluded if the results were not solely from HNSCC, mixed with various tumors, or if information for calculating hazard ratios (HR) and 95% confidence interval (95%CI) of outcomes was insufficient.

### Data extraction and quality assessment

Three authors (JKC, GJL and HSJ) independently identified the eligible articles and collected the following data: (i) publication information; the first author’s name, year of publication, country of study conducted, (ii) CTC data; sites of sample collection, sampling time point, methods and tumor markers to detect CTCs, (iii) clinical features; total patient numbers, number of CTC-positive patients, TNM stages, disease outcomes and follow-up duration. Any disagreements were resolved by discussion.

The quality of the studies was assessed with a risk of bias guideline, as described in the Cochrane reviewers’ Handbook 5.1.0.[17] In details, the adequacy of the following six categories was evaluated: (i) eligibility criteria, and (ii) exposure and outcome measurement, (iii) control of confounding factors, (iv) completeness of follow-ups, (v) presence of any suggestion of selective outcomes and (vi) presence of other high risk of bias. If all criteria above were met, the study was classified into low risk of bias group. If one or more criteria were inadequate, it was classified into high risk of bias group. One study was presented in the form of a meeting abstract. It did not report the exact duration of follow-up, which was considered as high risk of bias, although we could extract HR and 95%CI for outcomes depending on the status of CTC (S3 Table).[23]

### Statistical analyses

HRs with its 95%CI for each study was directly extracted from its study, or calculated using the available statistical information related to HR. Comparisons were made based on CTC status: the presence of CTC (positive CTC) versus absence of CTC (negative CTC). To determine the effect of CTC status on clinical outcomes, a pooled HR was estimated using the random effect model.[17,24] HR of more than 1 indicated worse outcome for the positive CTC group compared to the negative CTC group. The precision of estimates was quantified by 95%CI. Heterogeneity among studies was measured by Higgins and Green *I*^*2*^ statistics and Q-test.[17] The *I*^*2*^ ranged between 0% (no heterogeneity) and 100% (maximal heterogeneity), and the heterogeneity of the study was considered to be substantial at *P* of Q-test < 0.1 and *I*^*2*^ > 50%. We also evaluated the potential publication bias with Egger’s regression test for asymmetry and the funnel plot.[24]

Among the final enrolled studies, some had both multivariable adjusted HR and univariable HR values of CTC presence for survival outcomes, while others had only univariable HR results. Thus, the following analyses were also performed using only results of univariable HR and 95%CI, to confirm the results of main analyses using multivariable HR and 95%CI. All analyses were executed using R 3.2.3 (Vienna, Austria; http://www.R-project.org/) with a package of metafor. A two-sided *P* of less than 0.05 was considered statistically significant.

## Results

### Literature search

**Fig 1:**
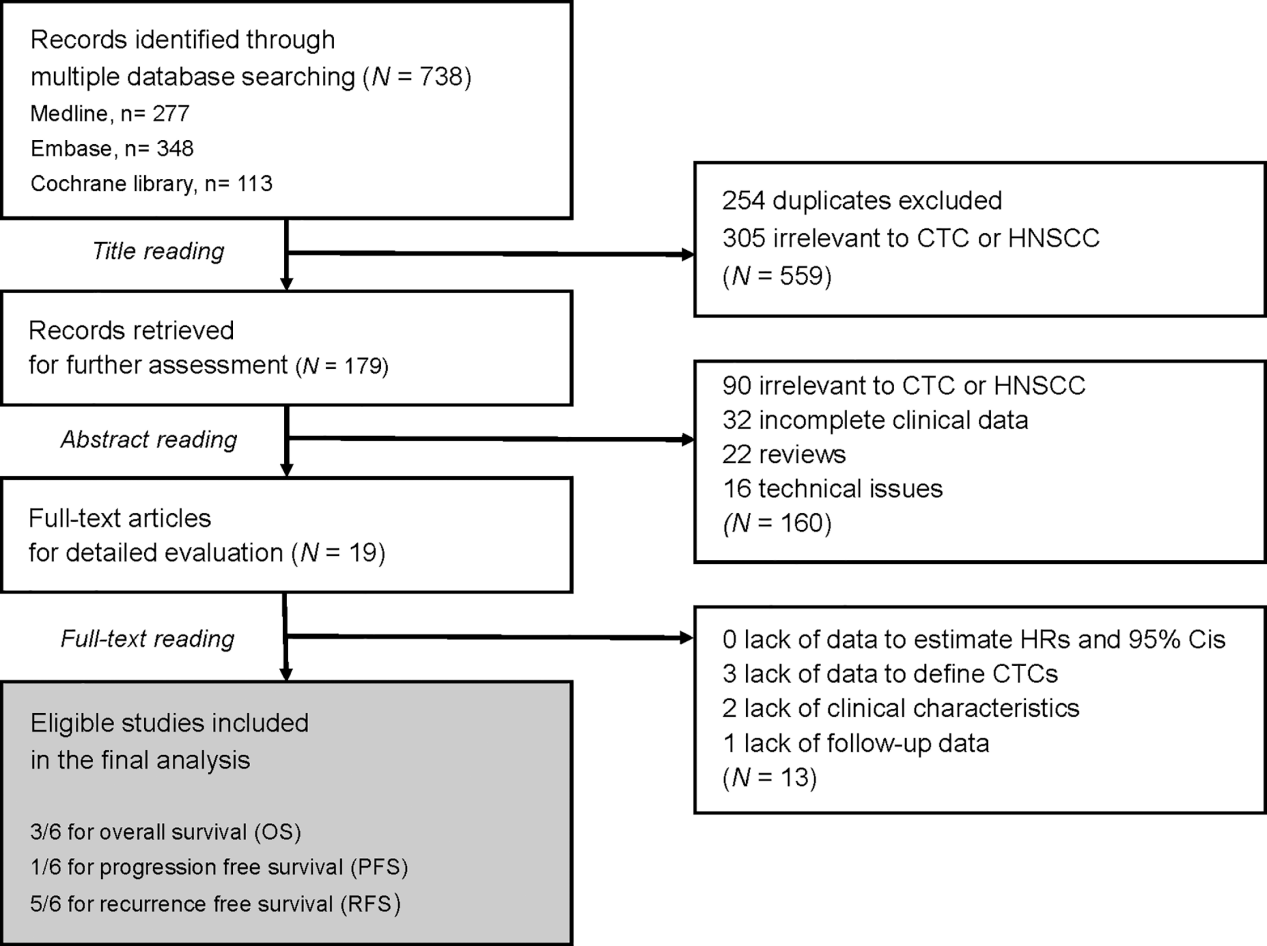
Flowchart of study selection process. CTC: circulating tumor cells, HNSCC: head and neck squamous cell carcinoma.

First, we identified 738 potentially relevant articles through multiple database searches. After the reviewing process, 719 studies were excluded (Fig 1). Among the remaining 19 studies, 13 were further excluded, mainly due to inability to estimate HR and 95%CI of outcomes, Thus, 6 studies were included in our meta-analysis; five full text articles [25–29] and one meeting abstract.[23]

Our enrolled studies were somewhat different from the studies of previous two meta-analyses.[15,16] Of note, we focused on different outcomes (RFS, PFS, OS) separately. Therefore, we excluded three study results which combined the outcomes of disease progression and recurrence or metastasis.[30–32] We additionally excluded one study from which we could not extract HR of outcomes according to the presence or absence of CTC.[33] In that study, the researchers had used CTC values as continuous variables in univariable analysis with different cutoff values (CTC = 22) (S4 Table).[33]

### Characteristics of identified studies

Characteristics of the included studies are listed in Table 1. A total of 429 patients were identified. The total number of patients ranged from 40 to 144 in individual studies. The sampling time of CTC in 4 studies was before the definite treatment, or during treatments in one study. One meeting abstract did not report the exact sampling time of CTC. CTCs were detected using RT-PCR, immunocytochemistry, flow cytometry (Raman scattering) and CellSearch system. The detected tumor markers mainly included epithelial cell adhesion molecule (EpCAM), cytokeratin, and epidermal growth factor receptor (EGFR). Four studies had multivariable adjusted HR of outcomes based on the presence or absence of CTC.[23,25,26,29] The remaining two had only univariable values.[27,28]

**Table 1:** Characteristics of studies included in the final analyses.

No.	Study (First Author)	Publication year	No. of subject	Detection method (Markers)	Sampling time	Stage	Tumor subsites	Follow-up	Outcome	Hazard ratio	Multivariate adjustment
1	Partridge[28]	2003	40	RT-PCR (E48) /ICC (Cytokeratin)	Baseline	M0	HNSCC	Median, 36 mo	OS, RFS	Reported	No
2	Jatana[27]	2010	48	ICC (Cytokeratin)	Baseline	NA	OC, Ophx, HPhx, Lx	Median, 19 mo	RFS	Reported	No
3	Gröbe[26]	2014	80	CellSearch (EpCAM, Cytokeratin)	Baseline	M0 = 75 M1 = 5	OC	Maximum, 10 YR	RFS	Reported	Yes
4	Grisanti[25]	2014	53	CellSearch (EpCAM, Cytokeratin)	Baseline	M0 = 26 M1 = 27	HNSCC	Median, 59 mo	OS, PFS	Reported	Yes
5	Tinhofer[29]	2014	144	RT-PCR (EGFR)	Mid-therapy	M0 = 144	OC, Ophx, HPhx, Lx	Median, 34 mo	OS, RFS	Reported	Yes
6	Wang[23]	2014	64	Flow cytometry^a^ (EpCAM)	NA	NA	HNSCC	NA	RFS	Reported	Yes
Total			429						OS: 3 PFS: 1 RFS: 5		

OS: overall survival, PFS: progression free survival, RFS: recurrence free survival; RT-PCR: Reverse transcription polymerase chain reaction, ICC, immunocytochemistry, EpCAM: epithelial cell adhesion molecule. M0: No distant metastasis at diagnosis, M1: Clinical distant metastasis, HNSCC: head and neck squamous cell carcinomas, OC: oral cavity, Ophx: oropharynx, Hphx: hypopharynx:, Lx: Larynx

^a^Flow cytometry with Raman scattering

### Prognostic significance of CTC and publication bias

**Fig 2:**
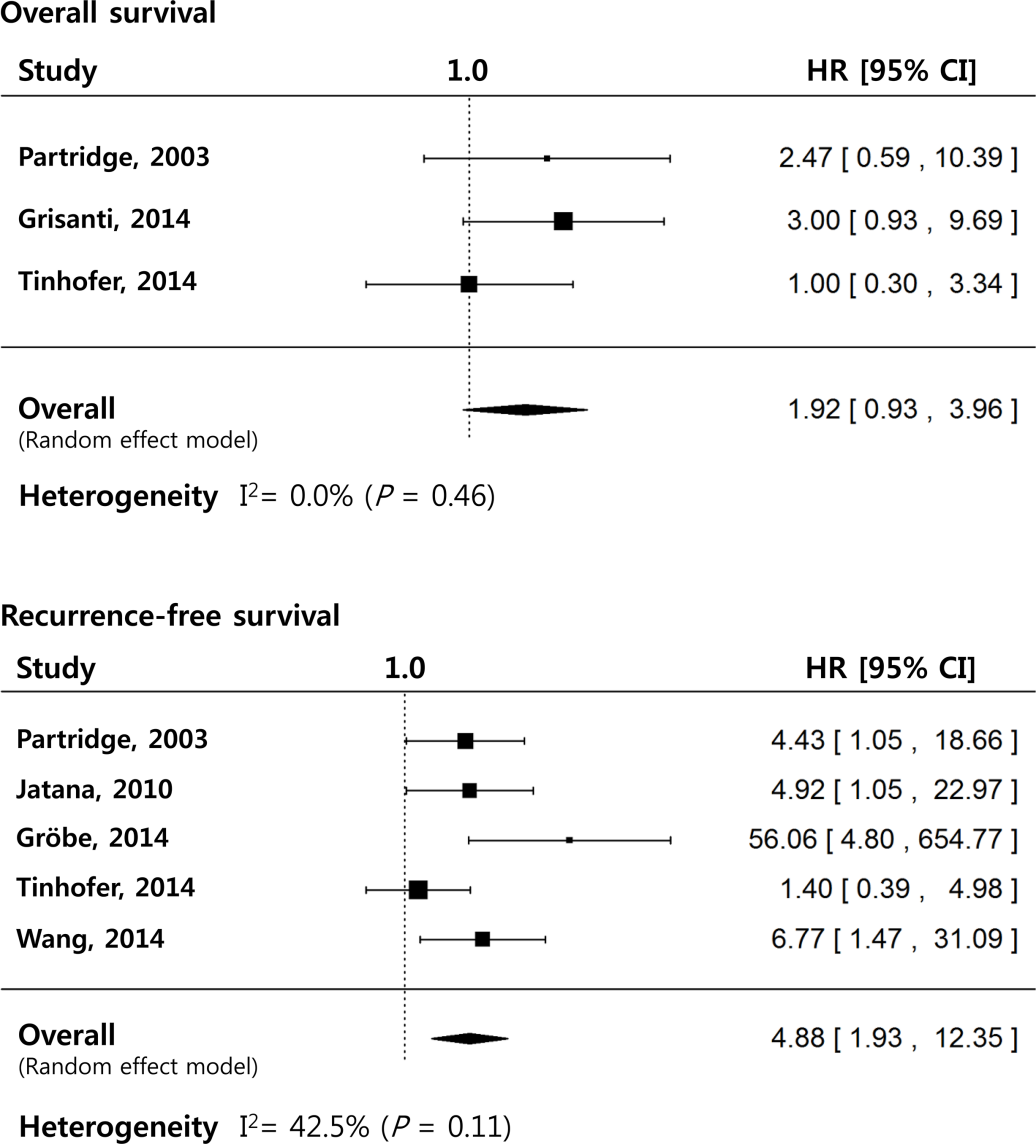
Forest plots illustrating prognostic value of CTC detection on the overall and recurrence-free survivals in HNSCC patients. HR: hazard ratio, 95%CI: 95% confidence interval.

Three eligible studies were analyzed in the OS meta-analysis, which revealed that a total HR of the random effect model was 1.92 [95%CI: 0.93–3.96] (*P* = 0.078), with very low heterogeneity (*I*^*2*^ = 0.0%, Q = 1.80 for 2 df, *P* = 0.41). Regarding the RFS meta-analysis, five studies were enrolled. An overall HR was 4.89 [95%CI: 1.93–12.35] (*P* < 0.001), indicating that the presence of CTC was a significant predictor for RFS in HNSCC patients. The heterogeneity of studies was not significant (*I*^*2*^ = 42.5%, Q = 7.57 for 4 df, *P* = 0.11) (Fig 2).

**Fig 3:**
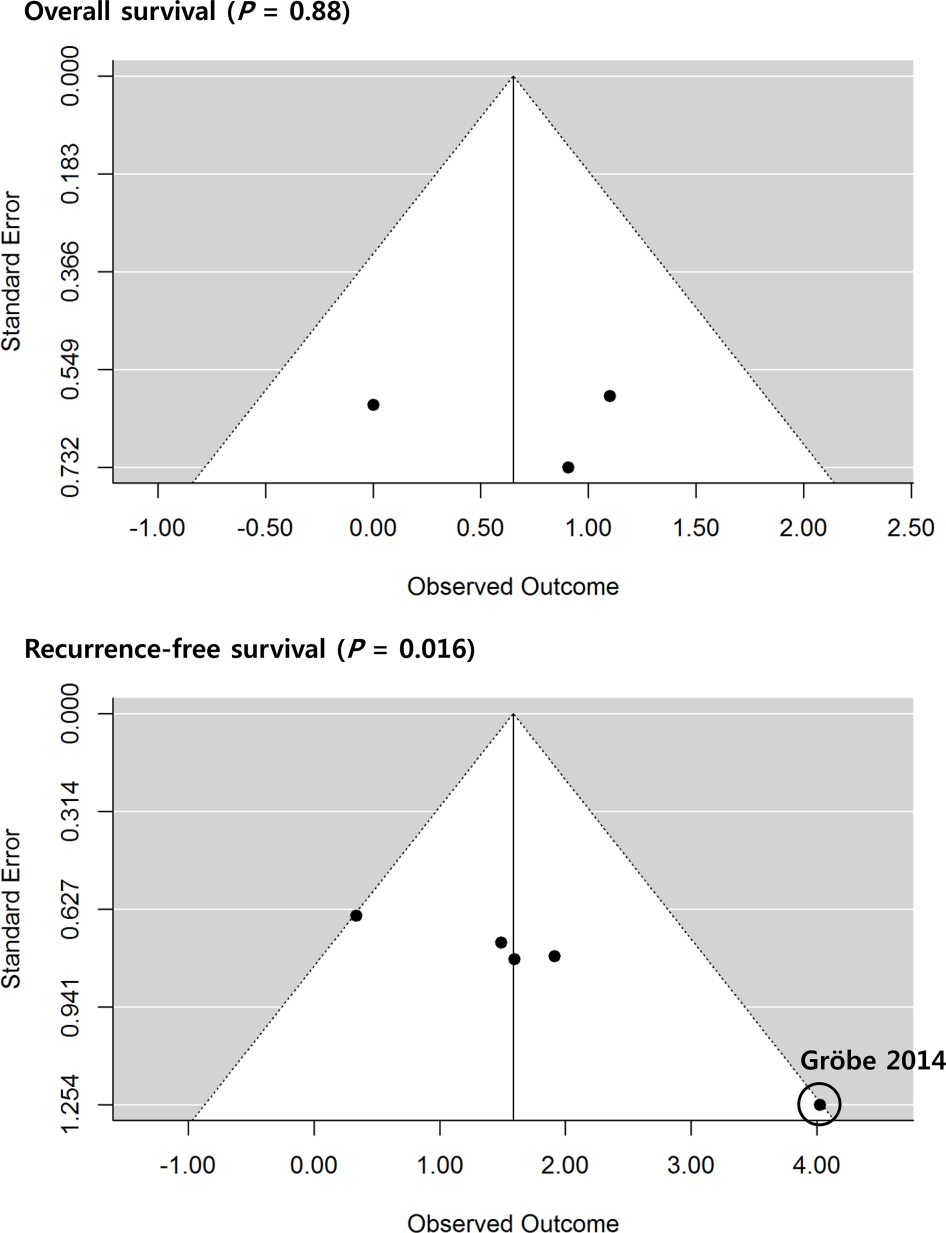
Funnel plots of analyzed studies according to the overall and recurrence-free survivals. SE: standard error, HR: hazard ratio.

Next, we evaluated publication bias by the Egger’s regression test and funnel plotting. In OS meta-analysis for the enrolled studies, the funnel plot showed a relatively symmetric distribution with Egger’s *P* vale of 0.881. However, there was a significant publication bias in RFS meta-analysis. Of note, one study showed relatively high multivariable HR value (HR = 56.06), compared to others. Egger’s *P* value was 0.016, indicating a substantial publication bias, mainly due to one extreme value (Fig 3).

Lastly, we performed additional analyses using univariable HRs and 95%CI of each study. In OS meta-analysis, the overall HR was 1.696 [95%CI: 0.83–3.45] (*P* = 0.145), which confirmed the primary results. The HR of the RFS meta-analysis was 3.80 [95%CI: 2.02–7.13] (*P* < 0.001), where the heterogeneity (*I*^*2*^ = 0.0%) and publication bias (Egger’s *P* = 0.33) were not significant. Overall, the following analyses produced similar values to those of primary analyses, suggesting that the results of the primary analyses were reliable (S1 Fig).

## Discussion

Compared to the two recent meta-analyses on CTCs of HNSCC,[15,16] our study has unique features, giving more comprehensive view on the prognostic value of CTC in HNSCC. The outcome in one of the previous studies was a combined event of recurrence and disease progression.[15,30–32] It also included a study of disseminated tumor cells from bone marrow biopsy.[34] Thus, it was hard to predict a potentially distinctive role of CTCs on loco-regional disease versus distant metastatic disease. The other meta-analysis addressed the prognostic significance of CTCs on disease-free, progression-free and overall survival, separately.[16] In agreement with our results, analyses of CTCs concerning PFS and OS showed no significant pooled HR, although the presence of CTC was significantly associated with shorter disease-free survival. However, our analyses enrolled more studies, which met the inclusion criteria, through meticulous electronic search including meeting abstract. Interestingly, some papers included in the previous meta-analyses did not met our criteria or give enough information to extract HRs and 95% CI,[33] which were excluded from our analyses (S4 Table). Thus, our analyses might be more comprehensive and reliable, and the following analyses using univariable values also supported our main findings.

RFS includes any recurrence (local, regional, or distant) and death due to any cause. PFS is defined as the time elapsed between treatment initiation and metastatic tumor progression or death from any cause.[20,21,35] Therefore, the main interest of disease with PFS is metastatic tumors in the distant sites in patients with systemic diseases at enrollment. Meanwhile, RFS focuses on disease recurrence from the status of no clinical tumors after curative treatments. In these analyses, we only identified one eligible study reporting PFS outcome,[25] thus we could not conduct a meta-analysis of PFS, separately.

Among the enrolled studies, there had been an issue in methodology of CTC detection (Table 1). Markers for CTC identification were inconsistent, although major markers were EpCAM and cytokeratin, except one with EGFR. Sampling time was also different; however, all studies had used peripheral blood for CTC measurement from patients before the completion of definite treatment, thus these CTC data appeared to reflect the initial tumor status in each patient. In addition, the subsites of HNSCC were not identical among the studies, which may affect the outcomes. Importantly, multivariable adjustments had been undertaken only in two thirds of the studies.

To overcome this study heterogeneity, we implemented three statistical methods for our meta-analyses. The first one was using a random effect model, rather than a fixed effect model for meta-analyses, to control for unobserved heterogeneity across the studies.[18] Next, we evaluated the degree of heterogeneity among the studies with Higgins *I*^*2*^ statistics and Q-test, and confirmed that the heterogeneity was not significant (*I*^*2*^ < 50% for OS and RFS). Lastly, we conducted additional analyses using univariable HRs for outcomes to validate the overall pooled HRs retrieved from mixed univariable and multivariable values. As a result of secondary analyses, we obtained the similar overall HRs, suggesting the primary results were reliable. Thus, our meta-analyses could give a more clear view of the prognostic significance of CTC in the pre-treatment settings of HNSCC, irrespective of specific CTC detection methods.

CTCs are tumor cells shed from primary tumors and metastatic deposits into the bloodstream.[10] Thus, total tumor burden might be related to the degree of CTCs. Particularly in HNSCC, local or regional recurrence is mainly induced by minimal residual tumor cells or biologically altered cells in the primary or regional sites.[36–38] However, whether CTCs directly contribute to the loco-regional recurrence remains unclear. Rather, high CTC counts may simply reflect big tumor burden at presentation, which has high risk of recurrence. In line with these assumptions, our data also confirmed that the presence of CTCs in the blood had the prognostic value in predicting RFS.

Distant metastases can develop from disseminated tumor cells via blood circulation. The high count of CTCs can be directly related to the emergence or growth of clinical distant metastatic foci. However, our results indicated that a simple presence of CTCs did not correlate with OS of HNSCC patients. The progression of systemic metastasis mainly determines OS of patients with HNSCC, and metastatic colonization and progression might require additional biological process from CTCs in the blood. Indeed, it has been reported that a unique subset of CTC, not the total count of CTC, can have a significant prognostic role predicting patients’ survival and metastatic progression.[33,39] Thus, we conclude that the presence of CTC itself would not be a good prognostic indicator for patients’ OS in HNSCC. This can be clarified by future meta-analysis of PFS.

The limitations of this study was the small number of enrolled studies, even though our meta-analysis identified the largest number of studies (*n* = 6) compared to previous meta-analyses. In the analyses of RFS, we also noted publication bias due to one outlier (HR = 56.06 from multivariable analysis),[26] which might have resulted in interpretational error. To adjust this potential bias, we further conducted a secondary analysis using univariable HR (HR = 5.73), which revealed the similar outputs (a pooled HR = 3.80 [95%CI 2.02–7.13], *I*^*2*^ = 0.00%) without publication bias (Egger’s *P* = 0.33).

## Conclusion

Our meta-analyses further support the view that the presence of CTC in the peripheral blood is not a significant prognostic indicator of OS in patients with HNSCC, although it could reflect the status of loco-regional disease outcomes.

## Supporting information

S1 Fig Forest plots and funnel plots illustrating prognostic value of CTC detection on overall survival and recurrence-free survival in HNSCC patients: Results of additional analyses using each univariable HR. (TIF)

S1 Table PRISMA checklist for this study. (PDF)

S2 Table Search strategy. (DOCX)

S3 Table Quality assessment of the included studies. (DOCX)

S4 Table Reasons for exclusion of studies, which were included in previous meta-analyses. (DOCX)

## References

[1] Kyzas PA, Evangelou E, Denaxa-Kyza D, Ioannidis JP. 18F-fluorodeoxyglucose positron emission tomography to evaluate cervical node metastases in patients with head and neck squamous cell carcinoma: a meta-analysis. J Natl Cancer Inst. 2008; 100:712–20. 10.1093/jnci/djn125 18477804

[2] Yao M, Dornfeld KJ, Buatti JM, Skwarchuk M, Tan H, Nguyen T, et al Intensity-modulated radiation treatment for head-and-neck squamous cell carcinoma—the University of Iowa experience. Int J Radiat Oncol Biol Phys. 2005; 63:410–21. 10.1016/j.ijrobp.2005.02.025 16168834

[3] Urken ML, Weinberg H, Buchbinder D, Moscoso JF, Lawson W, Catalano PJ, et al Microvascular free flaps in head and neck reconstruction. Report of 200 cases and review of complications. Arch Otolaryngol Head Neck Surg. 1994; 120:633–40. 8198786 10.1001/archotol.1994.01880300047007

[4] Jemal A, Siegel R, Xu J, Ward E. Cancer statistics, 2010. CA Cancer J Clin. 2010; 60:277–300. 10.3322/caac.20073 20610543

[5] Pignon JP, Bourhis J, Domenge C, Designe L. Chemotherapy added to locoregional treatment for head and neck squamous-cell carcinoma: three meta-analyses of updated individual data. MACH-NC Collaborative Group. Meta-Analysis of Chemotherapy on Head and Neck Cancer. Lancet. 2000; 355:949–55. 10768432

[6] Bonner JA, Harari PM, Giralt J, Azarnia N, Shin DM, Cohen RB, et al Radiotherapy plus cetuximab for squamous-cell carcinoma of the head and neck. N Engl J Med. 2006; 354:567–78. 10.1056/NEJMoa053422 16467544

[7] Chinn SB, Myers JN. Oral Cavity Carcinoma: Current Management, Controversies, and Future Directions. J Clin Oncol. 2015; 33:3269–76. 10.1200/JCO.2015.61.2929 26351335 PMC5320919

[8] Jatana KR, Lang JC, Chalmers JJ. Identification of circulating tumor cells: a prognostic marker in squamous cell carcinoma of the head and neck? Future Oncol. 2011; 7:481–4. 10.2217/fon.11.19 21463135 PMC4266857

[9] Steeg PS. Tumor metastasis: mechanistic insights and clinical challenges. Nat Med. 2006; 12:895–904. 10.1038/nm1469 16892035

[10] Pantel K, Speicher MR. The biology of circulating tumor cells. Oncogene. 2016; 35:1216–24. 10.1038/onc.2015.192 26050619

[11] Bidard FC, Fehm T, Ignatiadis M, Smerage JB, Alix-Panabieres C, Janni W, et al Clinical application of circulating tumor cells in breast cancer: overview of the current interventional trials. Cancer Metastasis Rev. 2013; 32:179–88. 10.1007/s10555-012-9398-0 23129208 PMC3655223

[12] Panteleakou Z, Lembessis P, Sourla A, Pissimissis N, Polyzos A, Deliveliotis C, et al Detection of circulating tumor cells in prostate cancer patients: methodological pitfalls and clinical relevance. Mol Med. 2009; 15:101–14. 10.2119/molmed.2008.00116 19081770 PMC2600498

[13] Ma XL, Xiao ZL, Liu L, Liu XX, Nie W, Li P, et al Meta-analysis of circulating tumor cells as a prognostic marker in lung cancer. Asian Pac J Cancer Prev. 2012; 13:1137–44. 22799295 10.7314/apjcp.2012.13.4.1137

[14] Jin T, Peng H, Wu H. Clinical value of circulating liver cancer cells for the diagnosis of hepatocellular carcinoma: A meta-analysis. Biomed Rep. 2013; 1:731–6. 10.3892/br.2013.139 24649019 PMC3916985

[15] Wang Z, Cui K, Xue Y, Tong F, Li S. Prognostic value of circulating tumor cells in patients with squamous cell carcinoma of the head and neck: a systematic review and meta-analysis. Med Oncol. 2015; 32:164 10.1007/s12032-015-0579-x 25895596

[16] Wu XL, Tu Q, Faure G, Gallet P, Kohler C, Bittencourt Mde C. Diagnostic and Prognostic Value of Circulating Tumor Cells in Head and Neck Squamous Cell Carcinoma: a systematic review and meta-analysis. Sci Rep. 2016; 6:20210 10.1038/srep20210 26831813 PMC4735798

[17] Higgins J, P,T, Green S. Cochrane Handbook for Systematic Reviews of Interventions Version 5.1.0 [updated March 2011]. Higggins J, P,T, Green S, editors: The Cochrane Collaboration; 2011.

[18] Tierney JF, Stewart LA, Ghersi D, Burdett S, Sydes MR. Practical methods for incorporating summary time-to-event data into meta-analysis. Trials. 2007; 8:16 10.1186/1745-6215-8-16 10.1186/1745-6215-8-16 17555582 PMC1920534

[19] Mathoulin-Pelissier S, Gourgou-Bourgade S, Bonnetain F, Kramar A. Survival end point reporting in randomized cancer clinical trials: a review of major journals. J Clin Oncol. 2008; 26:3721–6. 10.1200/JCO.2007.14.1192 10.1200/JCO.2007.14.1192 18669458

[20] Hudis CA, Barlow WE, Costantino JP, Gray RJ, Pritchard KI, Chapman JA, et al Proposal for standardized definitions for efficacy end points in adjuvant breast cancer trials: the STEEP system. J Clin Oncol. 2007; 25:2127–32. 10.1200/JCO.2006.10.3523 17513820

[21] Saad ED, Katz A. Progression-free survival and time to progression as primary end points in advanced breast cancer: often used, sometimes loosely defined. Ann Oncol. 2009; 20:460–4. 10.1093/annonc/mdn670 19095776

[22] Moher D, Liberati A, Tetzlaff J, Altman DG, Group P. Preferred reporting items for systematic reviews and meta-analyses: the PRISMA statement. Int J Surg. 2010; 8:336–41. 10.1016/j.ijsu.2010.02.007 20171303

[23] Wang X, Qian X, Beitler J, J., Zhang J, Kang H, Chen Z, et al. Evaluation prognostic significance of circulating tumor cells (CTCs) using multiplexed gold nanoparticles in patients with head and neck cancer [Abstract] In Proceedings of AACR Annual Meeting 2014. Cancer Res. 2014; 74:Abstract No. 4829. 10.1158/1538-7445

[24] Egger M, Davey Smith G, Schneider M, Minder C. Bias in meta-analysis detected by a simple, graphical test. BMJ. 1997; 315:629–34. 9310563 10.1136/bmj.315.7109.629 PMC2127453

[25] Grisanti S, Almici C, Consoli F, Buglione M, Verardi R, Bolzoni-Villaret A, et al Circulating tumor cells in patients with recurrent or metastatic head and neck carcinoma: prognostic and predictive significance. PLoS One. 2014; 9:e103918 10.1371/journal.pone.0103918 25105871 PMC4126745

[26] Grobe A, Blessmann M, Hanken H, Friedrich RE, Schon G, Wikner J, et al Prognostic relevance of circulating tumor cells in blood and disseminated tumor cells in bone marrow of patients with squamous cell carcinoma of the oral cavity. Clin Cancer Res. 2014; 20:425–33. 10.1158/1078-0432.CCR-13-1101 24218516

[27] Jatana KR, Balasubramanian P, Lang JC, Yang L, Jatana CA, White E, et al Significance of circulating tumor cells in patients with squamous cell carcinoma of the head and neck: initial results. Arch Otolaryngol Head Neck Surg. 2010; 136:1274–9. 10.1001/archoto.2010.223 21173379 PMC3740520

[28] Partridge M, Brakenhoff R, Phillips E, Ali K, Francis R, Hooper R, et al Detection of rare disseminated tumor cells identifies head and neck cancer patients at risk of treatment failure. Clin Cancer Res. 2003; 9:5287–94. 14614011

[29] Tinhofer I, Konschak R, Stromberger C, Raguse JD, Dreyer JH, Johrens K, et al Detection of circulating tumor cells for prediction of recurrence after adjuvant chemoradiation in locally advanced squamous cell carcinoma of the head and neck. Ann Oncol. 2014; 25:2042–7. 10.1093/annonc/mdu271 25057171

[30] Guney K, Yoldas B, Ozbilim G, Derin AT, Sarihan S, Balkan E. Detection of micrometastatic tumor cells in head and neck squamous cell carcinoma. A possible predictor of recurrences? Saudi Med J. 2007; 28:216–20. 17268699

[31] He S, Li P, He S, Long T, Zhang N, Fang J, et al Detection of circulating tumour cells with the CellSearch system in patients with advanced-stage head and neck cancer: preliminary results. J Laryngol Otol. 2013; 127:788–93. 10.1017/S0022215113001412 23835309

[32] Nichols AC, Lowes LE, Szeto CC, Basmaji J, Dhaliwal S, Chapeskie C, et al Detection of circulating tumor cells in advanced head and neck cancer using the CellSearch system. Head Neck. 2012; 34:1440–4. 10.1002/hed.21941 22076949

[33] Hsieh JC, Lin HC, Huang CY, Hsu HL, Wu TM, Lee CL, et al Prognostic value of circulating tumor cells with podoplanin expression in patients with locally advanced or metastatic head and neck squamous cell carcinoma. Head Neck. 2015; 37:1448–55. 10.1002/hed.23779 24844673

[34] Wollenberg B, Walz A, Kolbow K, Pauli C, Chaubal S, Andratschke M. Clinical relevance of circulating tumour cells in the bone marrow of patients with SCCHN. Onkologie. 2004; 27:358–62. 10.1159/000079088 15347890

[35] Burzykowski T, Buyse M, Piccart-Gebhart MJ, Sledge G, Carmichael J, Luck HJ, et al Evaluation of tumor response, disease control, progression-free survival, and time to progression as potential surrogate end points in metastatic breast cancer. J Clin Oncol. 2008; 26:1987–92. 10.1200/JCO.2007.10.8407 18421050

[36] Brennan JA, Mao L, Hruban RH, Boyle JO, Eby YJ, Koch WM, et al Molecular assessment of histopathological staging in squamous-cell carcinoma of the head and neck. N Engl J Med. 1995; 332:429–35. 10.1056/NEJM199502163320704 7619114

[37] Smits RW, Koljenovic S, Hardillo JA, Ten Hove I, Meeuwis CA, Sewnaik A, et al Resection margins in oral cancer surgery: Room for improvement. Head Neck. 2016; 38 Suppl 1:E2197–203. 10.1002/hed.24075 25899524

[38] Nathan CO, Franklin S, Abreo FW, Nassar R, De Benedetti A, Glass J. Analysis of surgical margins with the molecular marker eIF4E: a prognostic factor in patients with head and neck cancer. J Clin Oncol. 1999; 17:2909–14. 10.1200/JCO.1999.17.9.2909 10561370

[39] Weller P, Nel I, Hassenkamp P, Gauler T, Schlueter A, Lang S, et al Detection of circulating tumor cell subpopulations in patients with head and neck squamous cell carcinoma (HNSCC). PLoS One. 2014; 9:e113706 10.1371/journal.pone.0113706 25479539 PMC4257624

