# Supplementary figures and images for: Differential impact of circulating tumor cells on disease recurrence and survivals in patients with head and neck squamous cell carcinomas: An updated meta-analysis
Source: PLoS One. 2018 Sep 7;13(9):e0203758. doi: 10.1371/journal.pone.0203758 (PMC6128641; doi:10.1371/journal.pone.0203758)

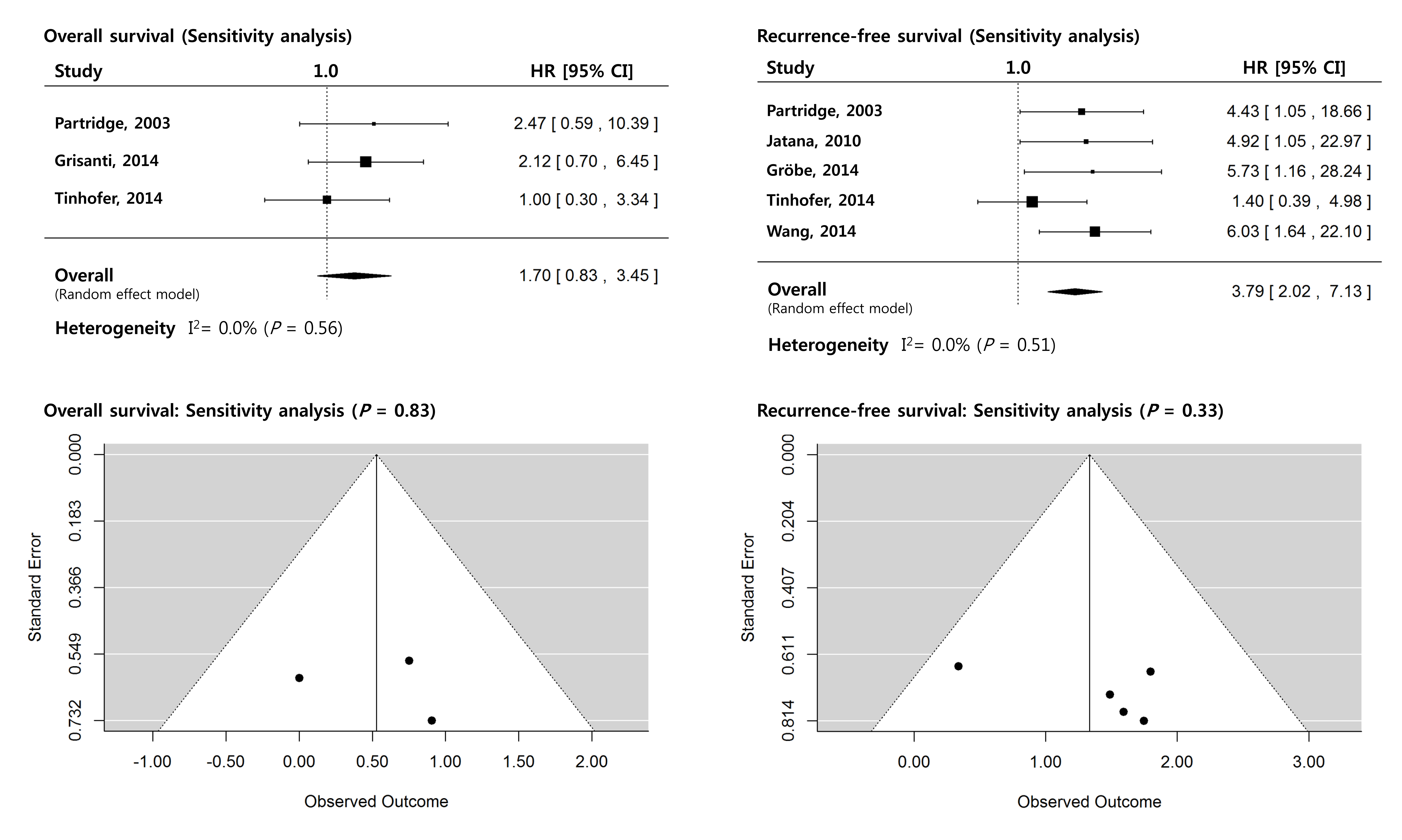

Supplement: S1 Fig — (TIF) [file pone.0203758.s001.tif]
