# Supplementary material for: Differential impact of circulating tumor cells on disease recurrence and survivals in patients with head and neck squamous cell carcinomas: An updated meta-analysis
Source: PLoS One. 2018 Sep 7;13(9):e0203758. doi: 10.1371/journal.pone.0203758 (PMC6128641; doi:10.1371/journal.pone.0203758)
Supplement: S2 Table — (DOCX) [file pone.0203758.s003.docx]

**S2 Table. Searching strategy**

Full electronic search strategy. The database search syntax listed below served as the basis for all search strategies. This search strategy was then adapted to the other electronic databases searched.

| Database: Medline, Embase and Cochrane library | |
| --- | --- |
| Date: December 31^st^, 2016 | |
|  | Query |
| #1 | Neoplasm* OR cancer* OR carcinoma* OR malignan* |
| #2 | “squamous cell carcinoma” OR “squamous cell” OR squamous OR SCC |
| #3 | “head and neck” OR HNSCC |
| #4 | Mouth OR oral OR “oral cavity” |
| #5 | Orophary* OR phary* OR tonsil* |
| #6 | Larynx OR larynx* |
| #7 | Hypophary* |
| #8 | Nasophary* |
| #9 | “nasal cavity” |
| #10 | “circulating tumor cell” OR “circulating tumor cells” OR CTC* |
| #11 | Prognos* OR outcome* |
| #12 | #1 OR #2 |
| #13 | #3 OR #4 OR #5 OR #6 OR #7 OR #8 OR #9 |
| #14 | #12 AND #13 |
| #15 | #10 AND #14 |
| #16 | #11 AND #15 |
