# Supplementary material for: Differential impact of circulating tumor cells on disease recurrence and survivals in patients with head and neck squamous cell carcinomas: An updated meta-analysis
Source: PLoS One. 2018 Sep 7;13(9):e0203758. doi: 10.1371/journal.pone.0203758 (PMC6128641; doi:10.1371/journal.pone.0203758)
Supplement: S3 Table — (DOCX) [file pone.0203758.s004.docx]

**S3 Table.** Quality assessment of the included studies

| No. | Study  (First Author,  Publication year) | Were adequate eligibility criteria developed and applied? | Was the measurement of both exposure and outcome adequate? | Was confounding adequately controlled for? | Was the follow-up complete and adequate in duration? | Are reports of the study free of suggestion of selective outcome reporting? | Was the study free of other problems that put it at a high risk of bias? | Risk of bias |
| --- | --- | --- | --- | --- | --- | --- | --- | --- |
| 1 | Partridge (2003) | Yes | Yes | Yes | Yes | Yes | Yes | Low |
| 2 | Jatana (2010) | Yes | Yes | Yes | Yes | Yes | Yes | Low |
| 3 | Gröbe (2014) | Yes | Yes | Yes | Yes | Yes | Yes | Low |
| 4 | Grisanti (2014) | Yes | Yes | Yes | Yes | Yes | Yes | Low |
| 5 | Tinhofer (2014) | Yes | Yes | Yes | Yes | Yes | Yes | Low |
| 6 | Wang^a^ (2014) | Yes | Yes | Yes | NA | Yes | NA | High |

Abbreviations:

NA: Not available

^a^Meeting abstract
