# Supplementary material for: Differential impact of circulating tumor cells on disease recurrence and survivals in patients with head and neck squamous cell carcinomas: An updated meta-analysis
Source: PLoS One. 2018 Sep 7;13(9):e0203758. doi: 10.1371/journal.pone.0203758 (PMC6128641; doi:10.1371/journal.pone.0203758)
Supplement: S4 Table — (DOCX) [file pone.0203758.s005.docx]

**S4 Table**. Reasons for exclusion of studies, which were included in previous meta-analyses

| Previous meta-analyses | No. | Study  (First Author) | Publication year | Causes of exclusion |
| --- | --- | --- | --- | --- |
| Wang Z, et al. (2015)[1] | 1 | Wollenberg[2] | 2004 | CTC profiles from bone marrow |
|  | 2 | Guney[3] | 2007 | Insufficient follow-up data, cannot extract the hazard ratio of RFS or PFS, respectively |
|  | 3 | Nichols[4] | 2012 | Insufficient follow-up data, cannot extract the hazard ratio of RFS or PFS, respectively |
|  | 4 | He[5] | 2013 | Cannot extract the hazard ratio of RFS or PFS, respectively |
| Wu XL, et al (2016)[6] | 5 | Hsieh[7] | 2015 | Cannot extract HR based on the presence or absence of CTC.  (Different cutoffs of CTC, CTC = 22) |

**References:**

**1.** Wang Z, Cui K, Xue Y, Tong F, Li S. Prognostic value of circulating tumor cells in patients with squamous cell carcinoma of the head and neck: a systematic review and meta-analysis. Med Oncol. 2015; 32:164. https://doi.org/10.1007/s12032-015-0579-x PMID: 25895596

**2.** Wollenberg B, Walz A, Kolbow K, Pauli C, Chaubal S, Andratschke M. Clinical relevance of circulating tumour cells in the bone marrow of patients with SCCHN. Onkologie. 2004; 27:358-62. https://doi.org/10.1159/000079088 PMID: 15347890

**3.** Guney K, Yoldas B, Ozbilim G, Derin AT, Sarihan S, Balkan E. Detection of micrometastatic tumor cells in head and neck squamous cell carcinoma. A possible predictor of recurrences? Saudi Med J. 2007; 28:216-20. PMID: 17268699

**4.** Nichols AC, Lowes LE, Szeto CC, Basmaji J, Dhaliwal S, Chapeskie C, et al. Detection of circulating tumor cells in advanced head and neck cancer using the CellSearch system. Head Neck. 2012; 34:1440-4. https://doi.org/10.1002/hed.21941 PMID: 22076949

**5.** He S, Li P, He S, Long T, Zhang N, Fang J, et al. Detection of circulating tumour cells with the CellSearch system in patients with advanced-stage head and neck cancer: preliminary results. J Laryngol Otol. 2013; 127:788-93. https://doi.org/10.1017/S0022215113001412 PMID: 23835309

**6.** Wu XL, Tu Q, Faure G, Gallet P, Kohler C, Bittencourt Mde C. Diagnostic and Prognostic Value of Circulating Tumor Cells in Head and Neck Squamous Cell Carcinoma: a systematic review and meta-analysis. Sci Rep. 2016; 6:20210. https://doi.org/10.1038/srep20210 PMID: 26831813

**7.** Hsieh JC, Lin HC, Huang CY, Hsu HL, Wu TM, Lee CL, et al. Prognostic value of circulating tumor cells with podoplanin expression in patients with locally advanced or metastatic head and neck squamous cell carcinoma. Head Neck. 2015; 37:1448-55. https://doi.org/10.1002/hed.23779 PMID: 24844673
